# Uses of the Pension Fund

**Published:** 1888-10-20

**Authors:** 


### USES OF THE PENSION FUND.

A signal example of the value and uses of the National Pension Fund for Nurses, 8, King Street, E.C., has occurred during the past few days. A lady of great energy and ability during her active years of service in certain London and provincial hospitals, who is now aged, ill, and helpless, has recently lost her savings, and so found herself bereft of means. Inquiries were made of two societies which grant pensions under certain conditions, when it was found that 1,800 votes must be obtained before a pension could be secured. Apart from the expense involved, some years must elapse before election to a pension under the most favourable circumstances. Hearing of the case, the following gentlemen have subscribed £261 5s., and have purchased her an immediate annuity in the Pension Fund of £30 per annum: Mr. Junius S. Morgan, £56 5s.; Mr. Leopold de Rothschild, £50; Mr. Alexander Henderson, £50; Messrs. Borthwick, £50; Mr. Arthur Anderson, £25; Mr. Panmure Gordon, £20; and Mr. E. F. Coates, £10. This is a practical instance of the value, not only of the Pension Fund to nurses, but of the publicity which the press have given to the Fund, by which those interested in nurses and their work have come to recognise how they can most promptly and beneficially show their practical sympathy in cases like the one under notice.

